# Characterizing Microglia Morphology in the Frontal Cortex of Pair-Bonded and Unpaired Prairie Voles (*Microtus ochrogaster*)

**DOI:** 10.3390/ijms26188966

**Published:** 2025-09-15

**Authors:** Tori Keefauver, Kyle L. Gobrogge

**Affiliations:** 1 Undergraduate Program in Neuroscience, College of Arts and Sciences, Boston University, Boston, MA 02215, USA; 2 Department of Psychological and Brain Sciences, Boston University, Boston, MA 02215, USA; 3 Department of Anesthesiology and Perioperative Medicine, Center for Neuroscience, University of Pittsburgh, Pittsburgh, PA 15261, USA; 4 Department of Psychiatry, Translational Neuroscience Program, Center for Neuroscience, University of Pittsburgh, Pittsburgh, PA 15261, USA; 5 Women’s, Gender, and Sexuality Studies Program, College of Arts and Sciences, Boston University, Boston, MA 02215, USA

**Keywords:** *Microtus ochrogaster*, prairie vole, microglia, morphology, sex differences, glia

## Abstract

*Microtus ochrogaster*, monogamous prairie voles, serve as translational animal models for studying monogamy and pair bonding. Microglia, the resident immune cells of the brain, are one of several cell types still poorly understood in non-classical animal models including prairie voles. Microglia are known to play mechanistic roles in mediating social behaviors using inflammatory signaling, but the relationship between microglia reactivity and pair bonding has not yet been investigated. The present study first developed a robust protocol for quantitative histological visualization of microglia in *Microtus ochrogaster*. Second, it investigated differences in microglia morphology, a reliable index of microglia reactivity and function, in pair-bonded vs. unpaired voles. Sections containing prefrontal cortex (PFC) and anterior cingulate cortex (ACC) were stained for ionized calcium-binding adaptor molecule I (Iba1) using immunohistochemistry (IHC). IHC results provided evidence for the successful use of murine histological protocols in prairie voles. Quantification results revealed a sexually dimorphic effect of pair bonding on microglia: somas were significantly larger in pair-bonded vs. unpaired females, and somas were significantly smaller in pair-bonded vs. unpaired males. Additionally, somas were significantly larger in unpaired males than females, with larger somas indicating higher microglia reactivity. While conclusions are limited due to the small sample size, results provide novel characterization of microglia morphology in the frontal cortex and elucidate how pair bonding may influence microglia function in a sexually dimorphic manner.

## 1. Introduction

Monogamous prairie voles, *Microtus ochrogaster*, are used as non-traditional animal models used to study the impacts of social monogamy and pair bonding in translational research [1,2]. In prairie voles, a pair bond is a lifelong bond with biological correlates that forms between two mating partners [1]. In humans, a pair bond is a lifelong bond with biological correlates that is created during long term romantic or sexual partnerships, long term friendships, and selective caregiver attachments [2,3,4]. The similarities between prairie vole pair bonds and human pair bonds, along with the importance of human relationships to social wellbeing, combine to make prairie voles an excellent model species for studying human behavior [5]. However, only a limited number of research groups currently study prairie voles due to their lack of commercial availability [6]. Prairie vole colonies can only be established via wild stock capture and progressive outbreeding [7], resulting in minimal and slow development of transgenic tools [8,9,10,11,12,13]. While prairie voles have been studied in certain laboratories for decades, their commercial unavailability combined with slow transgenic progress leaves prairie voles as understudied compared to other rodent model systems (e.g., mice and rats) [5].

Microglia are the resident immune cells of the brain, existing as the only macrophages in the brain parenchyma [14]. Microglia in the central nervous system support neurons and are important for cleanup of cellular debris, assistance with inflammatory responses, and pruning of synapses [15,16]. While microglia have been well studied in mice, rats, and humans for several decades [17], the first study of microglia in prairie voles did not occur until 2016 [18]. Thus, little is known about microglia in prairie voles [19].

Studies from the past decade have begun to investigate vole microglia but have focused on the effects of cohousing vs. isolation housing, not on the effects of pair bonding [20,21]. Isolated adult voles show differences in microgliosis when compared to cohoused voles, corresponding also to differences in depressive symptoms, neuronal activation and neurochemical expression [20]. These results were replicated in adolescent voles using a model of post-weaning isolation housing to demonstrate brain region specific changes in microglia density, an index of microgliosis [21]. Three additional studies have examined microglia morphology in response to endocrine disruption, partner loss, and chemical exposures [18,22,23]. However, no studies to date have investigated discrete differences in microglia morphology when comparing paired and unpaired (sexually naïve) voles.

Due to the lack of microglia research in prairie voles, findings from mouse, rat, and human postmortem research must be used to make predictions about prairie vole microglia histology [24]. Two basic classifications of microglia are homeostatic microglia and reactive microglia. Homeostatic microglia (often called “ramified”) are characterized by small triangular somas and many long, arborized processes. They function by contributing to several homeostatic functions such as surveillance, neurogenesis, and synapse monitoring and pruning [15,17]. In contrast, reactive microglia are characterized by larger, rounder somas with fewer and shorter processes [17]. Formerly referred to as “activated” microglia, reactive microglia are present during a brain’s inflammatory response and are localized in brain areas with high levels of inflammatory signaling. While the correlation between reduced branching and inflammation has long been assumed, a study by Madry et al. recently demonstrated a mechanistic link between reduced microglia branching and microglia cytokine release [25].

At the intersection of microglia in prairie voles as an understudied cell type and prairie voles as a socially monogamous model species lies the potential role of microglia in social behavior. The neuroplasticity of vole brains during pair bonding marks the pair bond as a key developmental timepoint when changes to social behavior may occur [5,26]. Microglia are also hypothesized to play distinct functional roles in mediating social behaviors [27,28,29,30]. The present study commences at this intersection by comparing microglia in pair-bonded prairie voles to microglia of unpaired prairie voles to elucidate microglia changes that occur in the presence of a biological pair bond.

The most common brain areas studied in prairie voles are the nucleus accumbens (NAc) and the amygdala (AG) [20,21]. These brain regions were identified as neuroanatomical sites of pair bonding via a comparative study of oxytocin receptor density in the brains of prairie voles versus non-monogamous, closely related vole species [31]. The five studies that have examined microglia morphology in prairie voles have all done so in the NAc and AG [18,20,21,22,23]. However, the same comparative study used to identify the NAc and AG as important brain regions for pair bonding also identified the prefrontal cortex (PFC) and anterior cingulate cortex (ACC) as equally important to the pair bonding process. Only two of the five studies that have examined microglia morphology in prairie voles have done so in the PFC and ACC [22,23]. The reason for the prairie vole field’s focus on the NAc and AG is unclear, but is an identified research gap by leaders in the field [19]. The PFC and ACC are uniquely involved in social information processing, especially in humans [32,33], whereas the NAc and AG are not involved in information processing but rather in mediating responses to social stimuli [34,35]. Extending the breadth of prairie vole research to include studies of the PFC and ACC yields unique information about how pair bonding information is processed, including the communications between cortical and limbic regions, that cannot be gained by studying the NAc and AG.

The present study uses quantitative immunohistochemistry (IHC) to characterize microglia morphology in the PFC and ACC of *Microtus ochrogaster,* and to elucidate the relationship between pair bonding status and microglia reactivity in prairie voles. We tested the hypothesis that there is a sexually dimorphic relationship between pair bonding status and microglia morphology. Our results, while preliminary, supported our hypothesis and made methodological contributions to the literature, supporting that murine IHC protocols can be successfully implemented in *Microtus ochrogaster* to histologically stain and quantify microglia morphology.

## 2. Results

### 2.1. Characterizing Microglia in Prairie Voles

**Figure 1:**
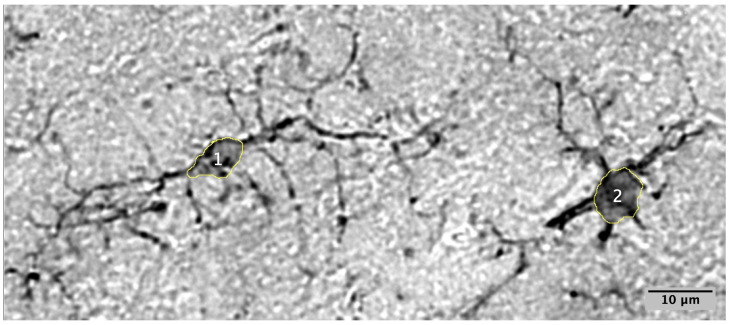
Homeostatic and reactive microglia. Photomicrograph of homeostatic (left) and reactive (right) microglia found next to each other in the vole prefrontal cortex (PFC). Image provides evidence for: (**1**) homeostatic phenotype with smaller soma (40 μ^2^), extensive arborized branching, and lower Iba1 stain intensity, and (**2**) reactive phenotype with larger soma (75 μm^2^), reduced branching, and higher Ionized calcium-binding adaptor molecule I (Iba1) stain intensity.

**Figure 2:**
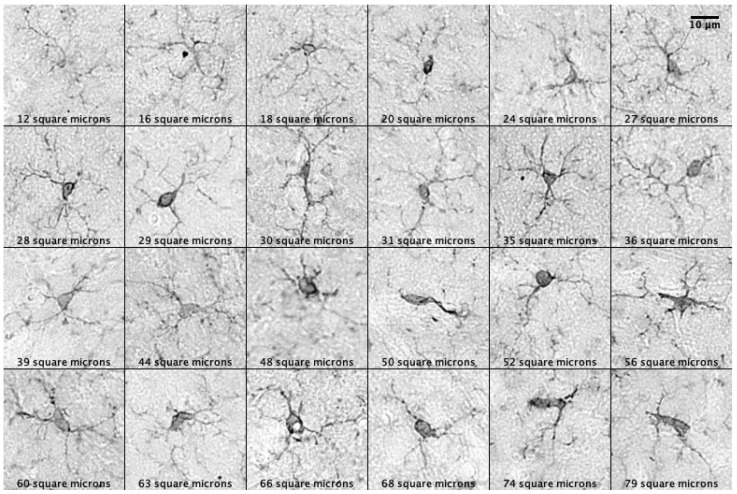
Montage of photomicrographs of increasing soma size. Images provides evidence that in prairie voles, branching decreases as the soma area and Iba1 stain intensity increase. Two-dimensional soma area is labeled on each image.

A total of 12,657 microglia were identified and their soma sizes measured. An area of 24.668 square millimeters (mm^2^) in the anterior cingulate cortex (ACC) and 13.756 mm^2^ in the prefrontal cortex (PFC) were analyzed for a total combined area of 38.424 mm^2^. Only somas which fell within the threshold of 10–80 square microns were counted and measured (See Materials and Methods for description of how threshold was determined). The existence of both homeostatic and reactive microglia in the vole tissue was confirmed **(**Figure 1). Photomicrographs of microglia across the entire soma threshold support the notion that in prairie voles, microglia with smaller somas contain longer and more numerous processes, while microglia with larger somas contain shorter and fewer processes (Figure 2).

Mean (M) microglia density across all animals and brain regions was 330.71 cells per mm^2^ (SD = 102; N = 228 images; *n* = 12,657 cells). Mean densities in females and males were 318.33 cells per mm^2^ (SD = 97; N = 116 images; *n* = 6281 cells) and 340.69 cells per mm^2^ (SD = 115; N = 112 images; *n* = 6376 cells), mean densities in unpaired and paired voles were 314.63 cells per mm^2^ (SD = 79; N = 110 images; *n* = 6142 cells) and 345.71 cells per mm^2^ (SD = 122; N = 118 images; *n* = 6515 cells), and densities in PFC and ACC regions were 341.23 cells per mm^2^ (SD = 95; N = 89 images; *n* = 4649 cells) and 323.98 cells per mm^2^ (SD = 106; N = 139 images; *n* = 8008 cells), respectively.

Regarding the experimental variables, the following mean densities represent measurements from the ACC and PFC combined: Paired Males (M = 344.16; SD = 132; N = 64 images; *n* = 3464 cells), Paired Females (M = 334.96; SD = 117; N = 54 images; *n* = 3051 cells), Unpaired Males (M = 328.90; SD = 86; N = 48 images; *n* = 2912 cells), Unpaired Females (M = 298.76; SD = 80; N = 62 images; *n* = 3230 cells).

The following mean densities represent measurements from the ACC only: Paired Males (M = 338.54; SD = 126; N = 37 images; *n* = 2162 cells), Paired Females (M = 333.54; SD = 137; N = 32 images; *n* = 1928 cells), Unpaired Males (M = 328.92; SD = 85; N = 35 images; *n* = 2286 cells), Unpaired Females (M = 285.37; SD = 68; N = 35 images; *n* = 1632 cells).

The following mean densities represent measurements from the PFC only: Paired Males (M = 351.59; SD = 141; N = 27 images; *n* = 1302 cells), Paired Females (M = 322.44; SD = 107; N = 22 images; *n* = 1123 cells), Unpaired Males (M = 305.35; SD = 125; N = 13 images; *n* = 626 cells), Unpaired Females (M = 327.19; SD = 69; N = 27 images; *n* = 1598 cells). Densities are reported for descriptive purposes, but testing for differences in cell density between groups was not performed.

Mean microglia soma area across all animals and brain regions was 29.69 μm^2^ (SD = 10.95; N = 228 images; *n* = 12,657 cells). Mean soma areas in females and males were 29.14 μm^2^ (SD = 10.51; N = 116 images; *n* = 6281 cells) and 30.40 μm^2^ (SD = 11.38; N = 112 images; *n* = 6376 cells), mean soma areas in unpaired and paired voles were 29.78 μm^2^ (SD = 10.99; N = 110 images; *n* = 6142 cells) and 29.62 μm^2^ (SD = 10.91; N = 118 images; *n* = 6515 cells), and soma areas in PFC and ACC regions were 29.56 μm^2^ (SD = 10.79; N = 89 images; *n* = 4649 cells) and 29.77 μm^2^ (SD = 11.05; N = 139 images; *n* = 8008 cells), respectively.

Regarding the experimental variables, the following soma areas represent measurements from the ACC and PFC combined: Paired Males (M = 29.38; SD = 10.81; N = 64 images; *n* = 3464 cells), Paired Females (M = 29.88; SD = 11.02; N = 54 images; *n* = 3051 cells), Unpaired Males (M = 31.72; SD = 11.76; N = 48 images; *n* = 2912 cells), Unpaired Females (M = 28.16; SD = 9.82; N = 62 images; *n* = 3230 cells).

The following mean densities represent measurements from the ACC only: Paired Males (M = 28.97; SD = 10.51; N = 37 images; *n* = 2162 cells), Paired Females (M = 29.75; SD = 11.13; N = 32 images; *n* = 1928 cells), Unpaired Males (M = 31.84; SD = 11.92; N = 35 images; *n* = 2286 cells), Unpaired Females (M = 28.09; SD = 9.95; N = 35 images; *n* = 1632 cells).

The following mean densities represent measurements from the PFC only: Paired Males (M = 30.04; SD = 11.26; N = 27 images; *n* = 1302 cells), Paired Females (M = 30.10; SD = 10.84; N = 22 images; *n* = 1123 cells), Unpaired Males (M = 30.93; SD = 11.95; N = 13 images; *n* = 626 cells), Unpaired Females (M = 28.23; SD = 9.69; N = 27 images; *n* = 1598 cells).

### 2.2. Evaluating the Normality of Microglia Soma Size Distributions in Prairie Voles

Since the expected distribution of microglia soma size was unknown, a quartile-quartile (Q-Q) plot and histogram were created to visualize the distribution of each of the four main variables: Unpaired Male Somas (*n* = 2), Unpaired Female Somas (*n* = 2), Paired Male Somas (*n* = 2), and Paired Female Somas (*n* = 2). These distributions were created for each of the four groups in three different brain areas: anterior cingulate cortex (ACC), prefrontal cortex (PFC), and both cortices combined into one group. The Q-Q plots and histograms were used to determine whether the data were normally distributed (Supplementary Figures S1–S6). All distributions displayed positive skewness. However, the distributions appeared to still be normally distributed, just shifted to the left, indicating that the lower half of the dataset may have been missing from the soma detection threshold. To quantify the significance of this skewed visualization, skewness and kurtosis were calculated and normality was quantified using chi-squared goodness of fit testing. These results are summarized in Supplementary Tables S1–S4. While the chi-squared normality testing indicated that all distributions are significantly unlikely to come from a normal distribution (*p* < 0.001), the skewness (s) and excess kurtosis (k) calculations did not indicate any significant levels of non-normality (−2 < s < +2; −2 < k < +2). Attempted transformations of the data using logarithmic (log10(x), log2(ex) and ln(x)), square-root (sqrt(x)), and reciprocal (1/x) transformations were unsuccessful. As a result, both parametric and non-parametric statistical tests were performed to account for the mixed results of model fit.

Parametric tests performed on the data included Independent Samples *t*-tests, One-Way Analysis of Variance (ANOVAs), and Multiple Comparison tests of means. The *t*-tests assumed unequal variance as indicated by the Q-Q plots in Supplementary Figures S1–S6. Results of parametric testing are summarized in Supplementary Tables S1–S5. Nonparametric tests performed on the data included Mann–Whitney U tests (Wilcoxon rank-sum tests), Kruskal–Wallis tests, and Multiple Comparison tests of medians (Supplementary Tables S6–S9).

Comparisons between the Independent Samples *t*-tests and Mann–Whitney U tests elucidate that the *t*-tests remained robust to any deviations from normality present in the data due to the large sample sizes. Both types of central-difference tests identified that the only comparison group with both a significant *p*-value and significant (or largest) effect size was Unpaired Males vs. Unpaired Females. This was true for the ACC (*p*_parametric_ < 0.001, d = 0.34, *p*_nonparametric_ < 0.001, median difference = 3.57), the PFC (*p*_parametric_ < 0.001, d = 0.25, *p*_nonparametric_ < 0.001, median difference = 1.56), and the combined regions (*p*_parametric_ < 0.001, d = 0.32, *p*_nonparametric_ < 0.001, median difference = 2.98).

Comparisons between the One-Way ANOVAs and Kruskal–Wallis tests elucidate that one-way ANOVA remained robust to deviations from normality present in the data due to the large sample sizes. Both types of one-way variance tests identified that group measures of central tendency were significantly different from one another. This was true for the ACC, PFC, and combined regions (*p*_parametric_ < 0.001, *p*_nonparametric_ < 0.001). Multiple comparison testing using the mean and median data from the ANOVAs and Kruskal–Wallis test revealed that the difference in means/medians of male paired and female paired microglia was not significant in any of the tested brain regions. All other possible group pairings showed at least one instance of significantly different means/medians.

To better understand the strong evidence for differences in the unpaired male and unpaired female groups produced by the multiple comparison tests, a three-way ANOVA was performed on the data (Supplementary Table S10). Although a three-way ANOVA assumes normality, the test was still considered valid due to the large sample size. The results produced evidence for a significant main effect of sex (*p* < 0.001) and a significant interaction effect of sex ×pairing status (*p* < 0.001) on microglia soma size. There was no evidence of main effects for pairing status or brain region. The interaction effects of pairing status × brain region and sex × brain region were not significant.

Simple linear regressions were performed to analyze the effect of age on mean soma area. Age did not significantly predict soma area in any brain region for any animal or group (Supplementary Figure S7): ACC only (r^2^ = 0.1002); PFC only (r^2^ = 0.05664); Combined regions (r^2^ = 0.08019).

### 2.3. The Effect of Pair Bonding on Microglia Morphology

**Figure 3:**
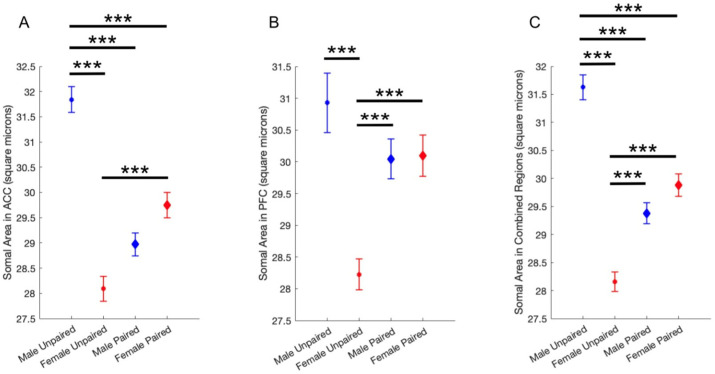
Pair bonding status affects microglia morphology in a sexually dimorphic manner. Results of multiple comparison tests from one-way analysis of variance (ANOVA). (**A**) Results in ACC. (**B**) Results in PFC. (**C**) Results in combined regions. *** *p* < 0.001. Blue indicates male subjects. Red indicates female subjects. Small circles indicate unpaired subjects. Large diamonds indicate paired subjects. Error bars = ±1 standard error of the mean.

Post hoc analyses revealed a significant effect of both sex and pairing status on microglia soma size. In both the ACC and the PFC, unpaired males had significantly larger somas than unpaired females. The difference between paired males and females was not significant. However, the somas of paired males and females were significantly different from the somas of unpaired males and females, but in opposite directions (Figure 3). The data suggests that pair bonding status affects microglia morphology in a sexually dimorphic manner.

## 3. Discussion

Robust sex differences in microglia reactivity are well-established in mice, rats, and humans [36,37]. Soma area can be used to measure microglia reactivity from histological images, as reactive microglia are characterized by large, round somas and few short processes. In the present study, analysis of over 12,000 microglia revealed a significant interaction between sex and pairing status with microglia reactivity in the ACC and PFC of prairie voles (Figure 3; Supplementary Table S10).

A robust array of both parametric and nonparametric statistical analyses indicated that somas in unpaired male voles were significantly larger than microglia in unpaired female voles in all animals (Figure 3; Supplementary Table S10). While the difference in soma size between unpaired males and unpaired females was the largest, there were also statistically significant differences between paired females and unpaired females, and between paired males and unpaired males. Females showed significant differences in both the ACC and PFC, whereas males showed significant differences in only the ACC. Results revealed no significant differences between paired female and paired male microglia in any brain region (Figure 3). Age was also considered as a confound but did not significantly predict mean soma area in any group (Supplementary Figure S7). Most importantly, there were no significant differences between all paired and all unpaired microglia: the differences only appeared when microglia were separated by sex (Supplementary Table S10).

Results from this brief report are the first to suggest that prairie vole microglia exhibit a sexually dimorphic morphology in both the ACC and PFC. This supports previous work which found evidence for a sexually dimorphic baseline of microglia morphology in the prairie vole PFC, cerebellum, and amygdala [23]. Additionally, it supports findings by Pohl et al. in which voles separated from their pair-bonded partner exhibited changes in microglia reactivity in the Paraventricular Nucleus (PVN), with sexual dimorphism emerging just four days after partner separation [22]. Taken together, this evidence for sexual dimorphism of microglia morphology in prairie voles can inform models of how social stress and isolation affect microglia in the monogamous prairie vole system.

This study has several limitations. First, due to the small sample size (*n* = 2/sex/group), the results discussed above should be considered preliminary results of this brief report, not decisive conclusions. Experiments need to be repeated with sufficiently powered groups to detect statistically significant differences and draw conclusions from them. However, regarding the present study, while the sample sizes were small, data were analyzed based on total cell counts (*n* = 12,657) and image counts (N = 228) for each group and variable, thus maximizing the sample sizes and statistical validity of the initial analyses.

Second, compared to non-monogamous rodents, the additional categorical variable of pair bonding status makes identifying the “control” group for prairie voles more challenging. While the present study included two possible pairing statuses for voles (unpaired (sexually naive) and paired), additional research utilizing a third group which has been separated after pair bonding is needed. Results from such a study would provide better framing for which pairing status should be considered the homeostatic control group for prairie voles.

Third, comparing differences in microglia morphology is not the most reliable way to characterize microglia’s functional state. Although microglia morphology is highly correlated with microglia function, morphological data is not to be used or interpreted in isolation. The technical and practical limitations of this brief report prevented the acquisition of functional measurements; thus, future research should use additional methods (e.g., additional IHC, Western blot, PCR, transcriptomics) to more accurately characterize the functional state of the microglia. Microglia studies in mice, rats, and humans achieve this by examining cytokine production or protein expression (e.g., microglia reactivity marker CD68) as direct indices of microglia function [27,38].

Findings from this study are only the fifth set of published experiments to successfully apply murine IHC protocols for Iba1 staining to prairie voles. Our research provides important methodological contributions to the field by documenting a protocol that is affordable and can be easily replicated by other scientists. Additionally, our study uses a different Iba1 antibody than the four previously published studies, broadening the scope of commercially produced antibodies that are documented for use in prairie voles. Future research should continue piloting murine antibodies and experimental protocols in prairie vole tissue to increase the accessibility and affordability of prairie vole research.

While the need for future expanded studies is recognized, the value of the present experiments and analyses persists, as the results provide evidence of a new successful IHC protocol and a preliminary framework for determining differences in frontal cortex microglia in paired versus unpaired prairie voles. The utility of using prairie voles to study diseases of social functioning cannot be understated; thus, future research should address the limitations of this brief report and continue working to overcome the methodological and empirical challenges of working with the prairie vole model system.

## 4. Materials and Methods

### 4.1. Animal Care

Adult prairie voles *(Microtus ochrogaster)* were laboratory-bred originating from systemic outbreeding of a wild stock captured near Champaign, Illinois. Sexually naïve male and female animals were group weaned at 21 ± 1 days and separated to group housing with same-sex siblings and age-matched same-sex non-siblings. Voles were maintained under a 12:12 h light-dark cycle in clear plastic cages (45 × 25 × 15 cm) with bedding and nesting material. Rooms were maintained at approximately 20 °C, and food and water were available ad libitum. A total of 8 adult prairie voles (2 pair-bonded females; 2 sexually naïve (unpaired) females; 2 pair-bonded males; 2 sexually naïve (unpaired) males) were used in this experiment. All voles were naïve to any previous experimentation or procedures. Subjects were 50–306 days of age at the start of the experiment. Experimenters were blinded to the vole pairing status during all portions of the experiment. This study did not include the use of humane endpoints. All procedures were conducted in accordance with the National Institutes of Health Guide and Use of Laboratory Animals and the Institutional Animal Care and Use Committees at the University of Kansas and the University of California, Davis.

### 4.2. Tissue Fixation and Sectioning

Prior to donation of the tissue to the authors, adult prairie voles were euthanized and transcardially perfused using 1× Phosphate-Buffered Saline (PBS) followed by 4% Paraformaldehyde (PFA). Brains were post-fixed in PFA at 4 degrees Celsius for storage and shipping to the authors. 30-micron coronal sections were obtained serially using a freezing microtome. Sections were stored in PBS with 0.05% sodium azide for long-term storage.

### 4.3. Immunohistochemistry

Due to prolonged storage in 4% PFA during storage and shipping, antigen retrieval was necessary to remove cross-linked formalins. Samples were incubated in 1× Tris-EDTA (TE) Buffer (Fisher BioReagents, Waltham, MA, USA, BP2477-500, pH 7.4) in a water bath at 95–100 degrees Celsius for 40 min. Samples were allowed to cool slowly back to room temperature while submerged in the TE Buffer before being moved to PBS for rinsing. Tissue carriers (Corning NetWell, Corning, NY, USA, CLS3479, 24 mm diameter, 74 μm mesh) six well plates, and paint brushes were used for free-floating immunohistochemistry (IHC). Sections were immunostained for Iba1 to label microglia. Reagents were used according to the Abcam Rabbit specific horseradish peroxidase (HRP)/diaminobenzidine (DAB) Detection IHC Kit (Cambridge, England, ab64261). Sections were washed in 1× PBS. Endogenous peroxidase activity was blocked with the hydrogen peroxide block in 0.3% Triton X-100 and PBS for 15 min at room temperature. Nonspecific binding was blocked with the Protein Block in 0.1% Triton-X 100 and PBS for 30 min at room temperature. Tissue was incubated in primary buffer containing Iba-1 polyclonal antibody (Thermo Fisher Invitrogen, Waltham, MA, USA, PA5-27436 Rabbit IgG) (1:500) for 48 h at 4 degrees Celsius and a secondary buffer containing biotinylated goat anti-rabbit IgG (H + L) (1:25) for 90 min at room temperature. Following incubation in the streptavidin peroxidase for one hour at room temperature, tissue was washed in sterile deionized water and moved to a solution of sterile deionized water containing a 1:25 dilution of 50× DAB Chromogen in DAB Substrate. Sections reacted with DAB for 10 min before being moved to a final rinse in PBS. Sections were mounted aqueously onto gel coated slides. Cover slips were placed using aqueous mounting medium (Abcam, Cambridge, England, AB64230) and glass coverslips.

### 4.4. Imaging and Quantification

Images of the ACC and PFC were captured on an LED microscope using 10× and 40x Leica Objectives, Basler camera (Ahrensburg, Germany, acA3088-57uc), and Basler imaging software (Version 4.1.0). Regions of Interest (ROIs) were determined using the Allen Mouse Brain Atlas to approximate vole brain regions. Microglia in the anterior cingulate cortex (ACC) and prefrontal cortex (PFC) were counted and traced using the “Analyze Particles” feature in Fiji/ImageJ (Version January 2024). PFC was considered as a composite region including the Prelimbic Areas and Infralimbic Areas. Particle threshold was determined to be 10–80 square microns by measuring the soma diameter of 15 random cells, determining the minimum and maximum diameter values, and taking the square of each. Data containing the soma area and ROI area for each image were exported and analyzed.

### 4.5. Statistical Analysis

All statistical analyses were completed using MATLAB R2023a. Independent samples *t*-tests, one-way ANOVAs, and two-way ANOVAs were completed to test for statistically significant differences in soma size between microglia in males and females and between microglia in paired and unpaired voles. Simple linear regressions were performed to determine whether vole age predicted mean soma area (Figure S7).

## Data Availability

The original data presented in the study are openly available in the author’s GitHub repository: VoleMicrogliaPaperData at https://github.com/torikeefauver/VoleMicrogliaPaperData (accessed on 14 September 2025).

## Supplementary Materials

The following supporting information can be downloaded at: https://www.mdpi.com/article/10.3390/ijms26188966/s1.

## Abbreviations

The following abbreviations are used in this manuscript:

PFC	Prefrontal Cortex
ACC	Anterior Cingulate Cortex
Iba1	Ionized calcium-binding adaptor molecule I
IHC	Immunohistochemistry
NAc	Nucleus Accumbens
AG	Amygdala
mm^2^	Square millimeters
μm^2^	Square microns (micrometers)
Q-Q	Quartile-quartile
s	Skewness
k	Kurtosis
ANOVA	Analysis of Variance
PVN	Paraventricular Nucleus
PBS	Phosphate-Buffered Saline
PFA	Paraformaldehyde
TE	Tris-EDTA
HRP	Horseradish Peroxidase
DAB	Diaminobenzidine
ROI	Region of Interest

## References

[1] Young K.A., Gobrogge K.L., Liu Y., Wang Z. (2011). The neurobiology of pair bonding: Insights from a socially monogamous rodent. Front. Neuroendocrinol..

[2] Aragona B.J., Wang Z. (2004). The prairie vole (*Microtus ochrogaster*): An animal model for behavioral neuroendocrine research on pair bonding. ILAR J..

[3] Gunnar M.R. (1998). Quality of early care and buffering of neuroendocrine stress reactions: Potential effects on the developing human brain. Prev. Med..

[4] Ditzen B., Schmidt S., Strauss B., Nater U.M., Ehlert U., Heinrichs M. (2008). Adult attachment and social support interact to reduce psychological but not cortisol responses to stress. J. Psychosom. Res..

[5] Badanes L.S., Dmitrieva J., Watamura S.E. (2012). Understanding Cortisol Reactivity across the Day at Child Care: The Potential Buffering Role of Secure Attachments to Caregivers. Early Child. Res. Q..

[6] Hiura L.C., Donaldson Z.R. (2023). Prairie vole pair bonding and plasticity of the social brain. Trends Neurosci..

[7] McGraw L.A., Young L.J. (2010). The prairie vole: An emerging model organism for understanding the social brain. Trends Neurosci..

[8] Loth M.K., Mesch K.T., Herrera-Garcia C., Brusman L.E., Donaldson Z.R. (2025). Lentiviral CRISPRa/i in the adult Prairie Vole Brain: Modulating Neuronal Gene Expression Without DNA Cleavage. Front. Genome Ed..

[9] Donaldson Z.R., Yang S.H., Chan A.W., Young L.J. (2009). Production of germline transgenic prairie voles (*Microtus ochrogaster*) using lentiviral vectors. Biol. Reprod..

[10] Keebaugh A.C., Modi M.E., Barrett C.E., Jin C., Young L.J. (2012). Identification of variables contributing to superovulation efficiency for production of transgenic prairie voles (*Microtus ochrogaster*). Reprod. Biol. Endocrinol..

[11] Horie K., Nishimori K. (2022). CRISPR/Cas9-Mediated Genetic Engineering to Generate a Disease Model Prairie Vole, Based on Species-Optimized Assisted Reproductive Technology. Methods Mol. Biol..

[12] McGraw L.A., Davis J.K., Lowman J.J., ten Hallers B.F., Koriabine M., Young L.J., de Jong P.J., Rudd M.K., Thomas J.W. (2010). Development of genomic resources for the prairie vole (*Microtus ochrogaster*): Construction of a BAC library and vole-mouse comparative cytogenetic map. BMC Genom..

[13] Horie K., Inoue K., Nishimori K., Young L.J. (2020). Investigation of Oxtr-expressing Neurons Projecting to Nucleus Accumbens using Oxtr-ires-Cre Knock-in prairie Voles (*Microtus ochrogaster*). Neuroscience.

[14] Li Q., Barres B.A. (2018). Microglia and macrophages in brain homeostasis and disease. Nat. Rev. Immunol..

[15] Sierra A., Paolicelli R.C., Kettenmann H. (2019). Cien Anos de Microglia: Milestones in a Century of Microglial Research. Trends Neurosci..

[16] Umpierre A.D., Wu L.J. (2020). Microglia Research in the 100th Year Since Its Discovery. Neurosci. Bull..

[17] Paolicelli R.C., Sierra A., Stevens B., Tremblay M.E., Aguzzi A., Ajami B., Amit I., Audinat E., Bechmann I., Bennett M. (2022). Microglia states and nomenclature: A field at its crossroads. Neuron.

[18] Rebuli M.E., Gibson P., Rhodes C.L., Cushing B.S., Patisaul H.B. (2016). Sex differences in microglial colonization and vulnerabilities to endocrine disruption in the social brain. Gen. Comp. Endocrinol..

[19] Loth M.K., Donaldson Z.R. (2021). Oxytocin, Dopamine, and Opioid Interactions Underlying Pair Bonding: Highlighting a Potential Role for Microglia. Endocrinology.

[20] Donovan M., Mackey C.S., Platt G.N., Rounds J., Brown A.N., Trickey D.J., Liu Y., Jones K.M., Wang Z. (2020). Social isolation alters behavior, the gut-immune-brain axis, and neurochemical circuits in male and female prairie voles. Neurobiol. Stress..

[21] Donovan M.L., Chun E.K., Liu Y., Wang Z. (2021). Post-weaning Social Isolation in Male and Female Prairie Voles: Impacts on Central and Peripheral Immune System. Front. Behav. Neurosci..

[22] Pohl T.T., Jung O., Di Benedetto B., Young L.J., Bosch O.J. (2021). Microglia react to partner loss in a sex- and brain site-specific manner in prairie voles. Brain Behav. Immun..

[23] Marinello W.P., Gillera S.E.A., Fanning M.J., Malinsky L.B., Rhodes C.L., Horman B.M., Patisaul H.B. (2022). Effects of developmental exposure to FireMaster(R) 550 (FM 550) on microglia density, reactivity and morphology in a prosocial animal model. Neurotoxicology.

[24] Leyh J., Paeschke S., Mages B., Michalski D., Nowicki M., Bechmann I., Winter K. (2021). Classification of Microglial Morphological Phenotypes Using Machine Learning. Front. Cell Neurosci..

[25] Madry C., Kyrargyri V., Arancibia-Carcamo I.L., Jolivet R., Kohsaka S., Bryan R.M., Attwell D. (2018). Microglial Ramification, Surveillance, and Interleukin-1beta Release Are Regulated by the Two-Pore Domain K(+) Channel THIK-1. Neuron.

[26] Shamay-Tsoory S.G., Marton-Alper I.Z., Markus A. (2024). Post-interaction neuroplasticity of inter-brain networks underlies the development of social relationship. iScience.

[27] Tay T.L., Bechade C., D’Andrea I., St-Pierre M.K., Henry M.S., Roumier A., Tremblay M.E. (2017). Microglia Gone Rogue: Impacts on Psychiatric Disorders across the Lifespan. Front. Mol. Neurosci..

[28] Kim H.J., Cho M.H., Shim W.H., Kim J.K., Jeon E.Y., Kim D.H., Yoon S.Y. (2017). Deficient autophagy in microglia impairs synaptic pruning and causes social behavioral defects. Mol. Psychiatry.

[29] Piirainen S., Chithanathan K., Bisht K., Piirsalu M., Savage J.C., Tremblay M.E., Tian L. (2021). Microglia contribute to social behavioral adaptation to chronic stress. Glia.

[30] Nelson L.H., Lenz K.M. (2017). Microglia depletion in early life programs persistent changes in social, mood-related, and locomotor behavior in male and female rats. Behav. Brain Res..

[31] Walum H., Young L.J. (2018). The neural mechanisms and circuitry of the pair bond. Nat. Rev. Neurosci..

[32] Eisenberger N.I. (2015). Meta-analytic evidence for the role of the anterior cingulate cortex in social pain. Soc. Cogn. Affect. Neurosci..

[33] Lai C.H. (2019). Promising Neuroimaging Biomarkers in Depression. Psychiatry Investig..

[34] Salgado S., Kaplitt M.G. (2015). The Nucleus Accumbens: A Comprehensive Review. Stereotact. Funct. Neurosurg..

[35] Mihara T., Mensah-Brown K., Sobota R., Lin R., Featherstone R., Siegel S.J. (2017). Amygdala activity associated with social choice in mice. Behav. Brain Res..

[36] Barko K., Shelton M., Xue X., Afriyie-Agyemang Y., Puig S., Freyberg Z., Tseng G.C., Logan R.W., Seney M.L. (2022). Brain region- and sex-specific transcriptional profiles of microglia. Front. Psychiatry.

[37] Bollinger J.L., Bergeon Burns C.M., Wellman C.L. (2016). Differential effects of stress on microglial cell activation in male and female medial prefrontal cortex. Brain Behav. Immun..

[38] Wolf S.A., Boddeke H.W., Kettenmann H. (2017). Microglia in Physiology and Disease. Annu. Rev. Physiol..

