# Supplementary material for: Characterizing Microglia Morphology in the Frontal Cortex of Pair-Bonded and Unpaired Prairie Voles (Microtus ochrogaster)
Source: Int J Mol Sci. 2025 Sep 15;26(18):8966. doi: 10.3390/ijms26188966 (PMC12469717; doi:10.3390/ijms26188966)
Supplement: Supplementary file 1 [file ijms-26-08966-s001.zip › ijms-3823863-supplementary.pdf]

Characterizing Microglia Morphology in the Frontal Cortex of Pair Bonded and Unpaired Prairie Voles *(Microtus ochrogaster)*

Supplementary Material

SUPPLEMENTARY TABLES S1-S10

SUPPLEMENTARY FIGURES S1-S7

SUPPLEMENTARY METHODS

TABLES AND FIGURES

Table S1

Descriptive Statistics of Distributions of Microglia Soma Areas

Sex	*Pairing Status*	*Brain Region*	*n*	*Skewness (s)*	*Excess Kurtosis (k)*	*p*	chi^2
Male							
	Unpaired						
		ACC	2,286	0.88	0.03	***$\mathbf{2.46 \times 10^{-46}}$	231.43
		PFC	626	1.06	0.35	***$\mathbf{6.01 \times 10^{-24}}$	122.06
		Both	2,912	0.92	0.09	***$\mathbf{1.97 \times 10^{-70}}$	344.38
	Paired						
		ACC	2,162	1.24	1.23	***$\mathbf{1.71 \times 10^{-92}}$	442.83
		PFC	1,302	1.06	0.53	***$\mathbf{1.37 \times 10^{-38}}$	191.26
		Both	3,464	1.17	0.94	***$\mathbf{3.26 \times 10^{-129}}$	613.23
Female							
	Unpaired						
		ACC	1,632	1.28	1.37	***$\mathbf{1.93 \times 10^{-54}}$	261.44
		PFC	1,598	1.35	1.72	***$\mathbf{1.58 \times 10^{-57}}$	275.81
		Both	3,230	1.31	1.54	***$\mathbf{4.79 \times 10^{-169}}$	797.72
	Paired						
		ACC	1,928	1.15	0.84	***$\mathbf{1.22 \times 10^{-64}}$	313.19
		PFC	1,123	1.02	0.47	***$\mathbf{1.23 \times 10^{-32}}$	163.22
		Both	3,051	1.11	0.71	***$\mathbf{1.29 \times 10^{-120}}$	578.06

Note. Positive skewness indicates moderate non-normality at values between +1 and +2, and significant non-normality at values greater than +2. Excess kurtosis was calculated assuming the kurtosis of a normal distribution to be 3. Excess kurtosis indicates significant non-normality at values greater than +2. *T*he reported *p* value is from a chi-squared test of normality. The chi-squared statistic is abbreviated *chi2stat.* ****p* < .001. *s* > +2. *k* > +2*

PARAMETRIC TESTING

Table S2

Independent Samples T-Tests: Microglia Soma Size in Vole Anterior Cingulate Cortex (ACC)

	n	*Mean*	*SD*	*t*	*p*	*df*	*Cohen's d*
Baseline (Unpaired) Sex Differences: ACC							
Unpaired Male	2,286	31.84	11.91	10.54	*** **1.29×10^{-24}**	3756.3	**0.34**
Unpaired Female	1,632	28.09	9.95				
Unpaired Male vs. Paired Male: ACC							
Unpaired Male	2,286	31.84	11.91	8.35	***8.94×10^{-17}**	4237.6	**0.25**
Paired Male	2,162	28.97	10.50				
Unpaired Female vs. Paired Female: ACC							
Unpaired Female	1,632	28.09	9.95	-4.71	***2.61×10^{-6}**	3560.4	-0.16
Paired Female	1,928	29.75	11.12				
Paired Sex Differences: ACC							
Paired Male	2,162	28.97	10.51	-2.29	***0.02**	3981.2	-0.07
Paired Female	1,928	29.75	11.13				
Unpaired vs. Paired: ACC							
Unpaired	3,918	30.23	11.27	3.54	***4.02×10^{-4}**	7768.1	0.08
Paired	4,090	29.35	10.81				

Note. SD = standard deviation. Cohen's *d* is considered significant at | *d* | > 0.2. Each subgroup of microglia (e.g. Unpaired Male) contains data combined from two subjects, with *n* indicating the sum of the total microglia identified in the ACC of those subjects. Mean soma area was measured in square microns and is shown for each subgroup of microglia. All t-tests assume unequal variance as indicated by Q-Q plots in Figures 1-2. *$p < .05$. ***$p < .001$.

Table S3

Independent Samples T-Tests: Microglia Soma Size in Vole Prefrontal Cortex (PFC)

	n	*Mean*	*SD*	*t*	*p*	*df*	*Cohen's d*
Baseline (Unpaired) Sex Differences: PFC							
Unpaired Male	626	30.93	11.95	5.13	***$\mathbf{3.44 \times 10^{-7}}$**	1021.6	**0.25**
Unpaired Female	1,598	28.23	9.69				
Unpaired Male vs. Paired Male: PFC							
Unpaired Male	626	30.93	11.95	1.57	0.1157	1241.0	0.08
Paired Male	1,302	30.04	11.26				
Unpaired Female vs. Paired Female: PFC							
Unpaired Female	1,598	28.23	9.69	-4.63	***$\mathbf{3.87 \times 10^{-6}}$**	2240.3	-0.18
Paired Female	1,123	30.10	10.84				
Paired Sex Differences: PFC							
Paired Male	1,302	30.04	11.26	-0.12	0.90	2393.9	-0.005
Paired Female	1,123	30.10	10.84				
Unpaired vs. Paired: PFC							
Unpaired	2,224	29.01	10.47	-3.36	***$\mathbf{7.87 \times 10^{-4}}$**	4673.3	-0.10
Paired	2,425	30.07	11.06				

Note. SD = standard deviation. Cohen's *d* is considered significant at | *d* | > 0.2. Each subgroup of microglia (e.g. Unpaired Male) contains data combined from two subjects, with *n* indicating the sum of the total microglia identified in the PFC (Infralimbic and Prelimbic Areas) of those subjects. Mean soma area was measured in square microns and is shown for each subgroup of microglia. All t-tests assume unequal variance as indicated by Q-Q plots in Figures 3-4. $^{*}p < .05$. $^{***}p < .001$.

Table S4

Independent Samples T-Tests: Microglia Soma Size in Combined Regions

	n	*Mean*	*SD*	*t*	*p*	*df*	*Cohen's d*
Baseline (Unpaired) Sex Differences: Combined Regions							
Unpaired Male	2,912	31.63	11.93	12.24	*****5.60 x 10^{-34}**	5470.3	**0.32**
Unpaired Female	3,230	28.16	9.82				
Unpaired Male vs. Paired Male: Combined Regions							
Unpaired Male	2,912	31.63	11.93	9.60	*****1.23 x 10^{-21}**	5504.3	**0.24**
Paired Male	3,464	28.97	10.51				
Unpaired Female vs. Paired Female: Combined Regions							
Unpaired Female	3,230	28.16	9.82	-6.53	*****7.27 x 10^{-11}**	6127.8	-0.16
Paired Female	3,051	29.88	11.02				
Paired Sex Differences: Combined Regions							
Paired Male	3,464	28.97	10.51	-3.54	*****4.05x 10^{-4}**	6419.5	-0.08
Paired Female	3,051	29.88	11.02				
Unpaired vs. Paired: Combined Regions							
Unpaired	6,142	29.78	10.99	0.81	0.42	12462	0.01
Paired	6,515	29.62	10.91				

Note. SD = standard deviation. Cohen's *d* is considered significant at | *d* | > 0.2. Each subgroup of microglia (e.g. Unpaired Male) contains data combined from two subjects, with *n* indicating the sum of the total microglia identified in the PFC (Infralimbic and Prelimbic Areas) and ACC combined of those subjects. Mean soma area was measured in square microns and is shown for each subgroup of microglia. All t-tests assume unequal variance as indicated by Q-Q plots in Figures 5-6. **p < .05.* ****p < .001.*

Table S5

One-way ANOVA Summary Table for Microglia Soma Area

Source	*df*	*MS*	*F*	*p*
ACC Groups	3	5057.41	42.20	***$\mathbf{4.80 \times 10^{27}}$
Within ACC Groups	7858	120.08		
ACC Total	7861			
PFC Groups	3	1565.32	13.54	***$\mathbf{8.50 \times 10^{9}}$
Within PFC Groups	4673	115.70		
PFC Total	4676			
Combined ACC and PFC Groups	3	6197.18	52.31	***$\mathbf{1.37 \times 10^{33}}$
Within Combined Groups	12535	118.48		
ACC and PFC Combined Total	12538			

Note. MS = Mean squares. *$*p < .05$. ***$p < .001$. The four variable groups for each brain region are the same: Unpaired Male, Unpaired Female, Paired Male, Paired Female.*

NONPARAMETRIC TESTING

Table S6

Mann-Whitney U tests: Microglia Soma Size in Vole Anterior Cingulate Cortex (ACC)

	n	*Median*	*U*	*p*	*Effect Size*
		Baseline (Unpaired) Sex Differences: ACC			
Unpaired Male	2,286	28.93	4,440,160	***$\mathbf{2.03 \times 10^{-22}}$	**3.57**
Unpaired Female	1,632	25.36			
		Unpaired Male vs. Paired Male: ACC			
Unpaired Male	2,286	28.93	4,959,936	***$\mathbf{3.87 \times 10^{-15}}$	2.75
Paired Male	2,162	26.18			
		Unpaired Female vs. Paired Female: ACC			
Unpaired Female	1,632	25.36	2,792,961	***$\mathbf{3.82 \times 10^{-5}}$	-1.41
Paired Female	1,928	26.78			
		Paired Sex Differences: ACC			
Paired Male	2,162	26.18	4,238,700	0.07	-0.60
Paired Female	1,928	26.7778			
		Unpaired vs. Paired: ACC			
Unpaired	3,918	27.30	15,262,000	**0.001	0.81
Paired	4,090	26.48			

Note. U = Mann-Whitney U-test statistic, the number of times a *y* precedes an *x* in an ordered arrangement of the elements in the two independent samples X and Y. *U* is reported for X only since $N_x \neq N_y$. *Effect Size* = Median(X) - Median(Y). Each subgroup of microglia (e.g. Unpaired Male) contains data combined from two subjects, with *n* indicating the sum of the total microglia identified in the ACC of those subjects. Median soma area was measured in square microns and is shown for each subgroup of microglia. **p < .05. **p< .01. ***p < .001.*

Table S7

Mann-Whitney U tests: Microglia Soma Size in Vole Prefrontal Cortex (PFC)

	n	*Median*	*U*	*p*	*Effect Size*
Baseline (Unpaired) Sex Differences: PFC					
Unpaired Male	626	27.48	794,790	*****3.40 x 10^{-5}**	**1.56**
Unpaired Female	1,598	25.92			
Unpaired Male vs. Paired Male: PFC					
Unpaired Male	626	27.48	656,022	0.17	0.41
Paired Male	1,302	27.07			
Unpaired Female vs. Paired Female: PFC					
Unpaired Female	1,598	25.92	2,092,164	*****4.14 x 10^{-5}**	-1.15
Paired Female	1,123	27.07			
Paired Sex Differences: PFC					
Paired Male	1,302	27.07	1,567,450	0.49	0
Paired Female	1,123	27.07			
Unpaired vs. Paired: PFC					
Unpaired	2,224	26.256	5,132,700	**0.003	-0.81
Paired	2,425	27.074			

Note. U = Mann-Whitney U-test statistic, the number of times a *y* precedes an *x* in an ordered arrangement of the elements in the two independent samples X and Y. *U* is reported for X only since $N_x \neq N_y$. *Effect Size* = Median(X) - Median(Y). Each subgroup of microglia (e.g. Unpaired Male) contains data combined from two subjects, with *n* indicating the sum of the total microglia identified in the PFC (Infralimbic and Prelimbic Areas) of those subjects. Median soma area was measured in square microns and is shown for each subgroup of microglia. **p < .05. **p< .01. ***p < .001.*

Table S8

Mann-Whitney U tests: Microglia Soma Size in Combined Regions

	n	*Median*	*U*	*p*	*Effect Size*
Baseline (Unpaired) Sex Differences: Combined Regions					
Unpaired Male	2,912	28.64	9,277,410	*****3.81×10^{-28}**	**2.98**
Unpaired Female	3,230	25.66			
Unpaired Male vs. Paired Male: Combined Regions					
Unpaired Male	2,912	28.64	9,321,200	*****2.13×10^{-13}**	2.16
Paired Male	3,464	26.48			
Unpaired Female vs. Paired Female: Combined Regions					
Unpaired Female	3,230	25.66	9,768,900	*****2.12×10^{-8}**	-1.26
Paired Female	3,051	26.93			
Paired Sex Differences: Combined Regions					
Paired Male	3,464	26.48	10,962,332	0.07	-0.45
Paired Female	3,051	26.93			
Unpaired vs. Paired: Combined Regions					
Unpaired	6,142	26.78	38,103,366	0.40	0.074
Paired	6,515	26.70			

Note. U = Mann-Whitney U-test statistic, the number of times a *y* precedes an *x* in an ordered arrangement of the elements in the two independent samples X and Y. *U* is reported for X only since $N_x \neq N_y$. *Effect Size* = Median(X) - Median(Y). Each subgroup of microglia (e.g. Unpaired Male) contains data combined from two subjects, with *n* indicating the sum of the total microglia identified in the PFC (Infralimbic and Prelimbic Areas) and ACC combined of those subjects. Median soma area was measured in square microns and is shown for each subgroup of microglia. **p < .05. **p< .01. ***p < .001.*

Table S9

Kruskal-Wallis Test of One-way Variance Summary Table for Microglia Soma Area

Source	*df*	*MS*	*chi-sq*	*p*
ACC Groups	3	1.88×10^{8}	109.50	***$\mathbf{1.40 \times 10^{-23}}$
Within ACC Groups	7858	5.08×10^{6}		
ACC Total	7861			
PFC Groups	3	1.61×10^{7}	26.45	***$\mathbf{7.66 \times 10^{-6}}$
Within PFC Groups	4673	1.81×10^{6}		
PFC Total	4676			
Combined ACC and PFC Groups	3	5.44×10^{8}	124.47	***$\mathbf{8.40 \times 10^{-27}}$
Within Combined Groups	12535	1.30×10^{7}		
ACC and PFC Combined Total	12538			

Note. MS = Mean squares. *$p < .05$. ***$p < .001$. *The four variable groups for each brain region are the same: Unpaired Male, Unpaired Female, Paired Male, Paired Female*

INTERACTION EFFECTS

Table S10

Three-way ANOVA Summary Table for Microglia Soma Area

Source	*df*	*MS*	*F*	*p*
Sex	1	1,017,000.6	47.51	***0.00
Pairing Status	1	8,307.3	0.39	0.53
Brain Region	1	26,402.3	1.23	0.27
Sex x Pairing Status	1	2,060,294.6	96.24	***0.00
Sex x Brain Region	1	622.6	0.03	0.86
Pairing Status x Brain Region	1	126,351.9	5.90	*0.02
Within Groups	12532	21,406.8		
Total	12538			

Note. MS = Mean squares. **p < .05. ***p < .001.*

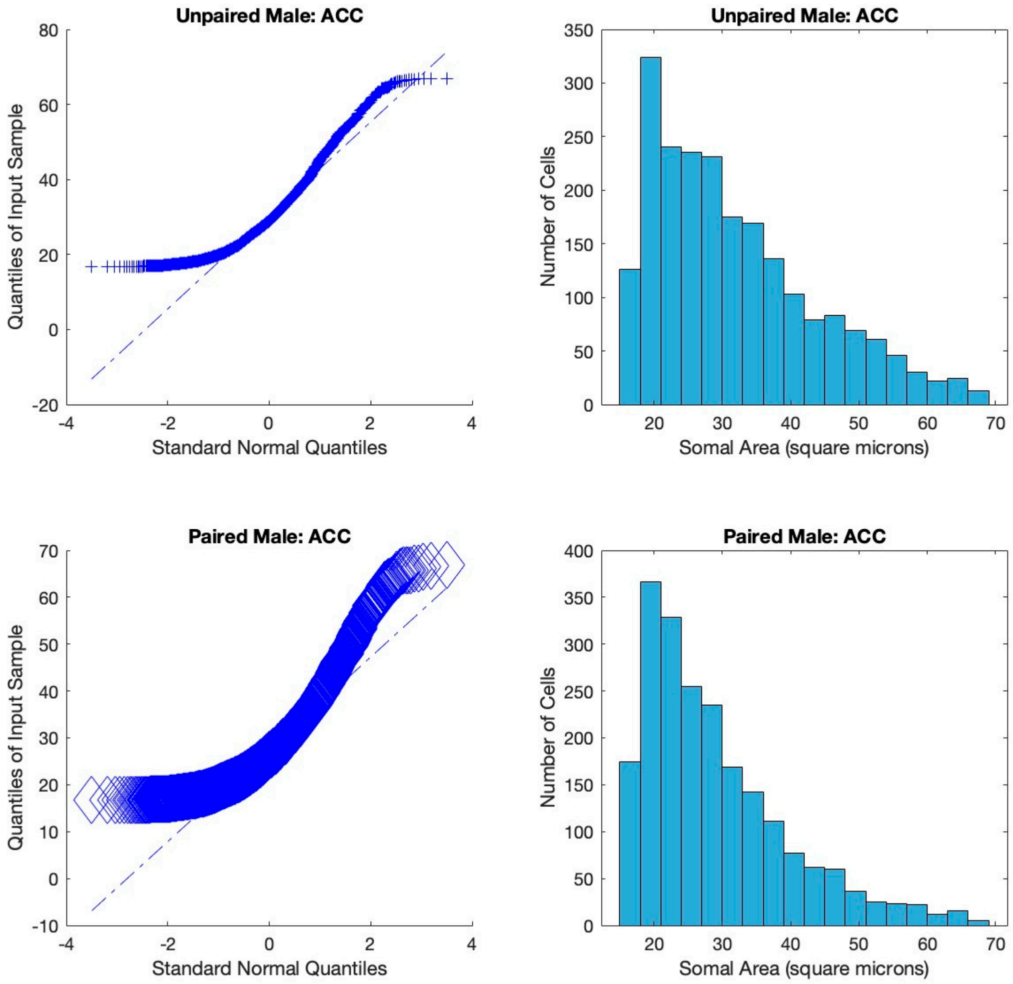

Figure S1. Male ACC distribution visualizations. Q-Q Plots (Left) and Histograms (Right) produced as visualizations of the distributions of microglia soma areas in the anterior cingulate cortex (ACC). Q-Q Plots depict the unequal variance of each distribution compared to a normal distribution as indicated by the diagonal dashed line. The color *blue* indicates male microglia. A small cross indicates unpaired microglia. A large diamond indicates paired microglia.

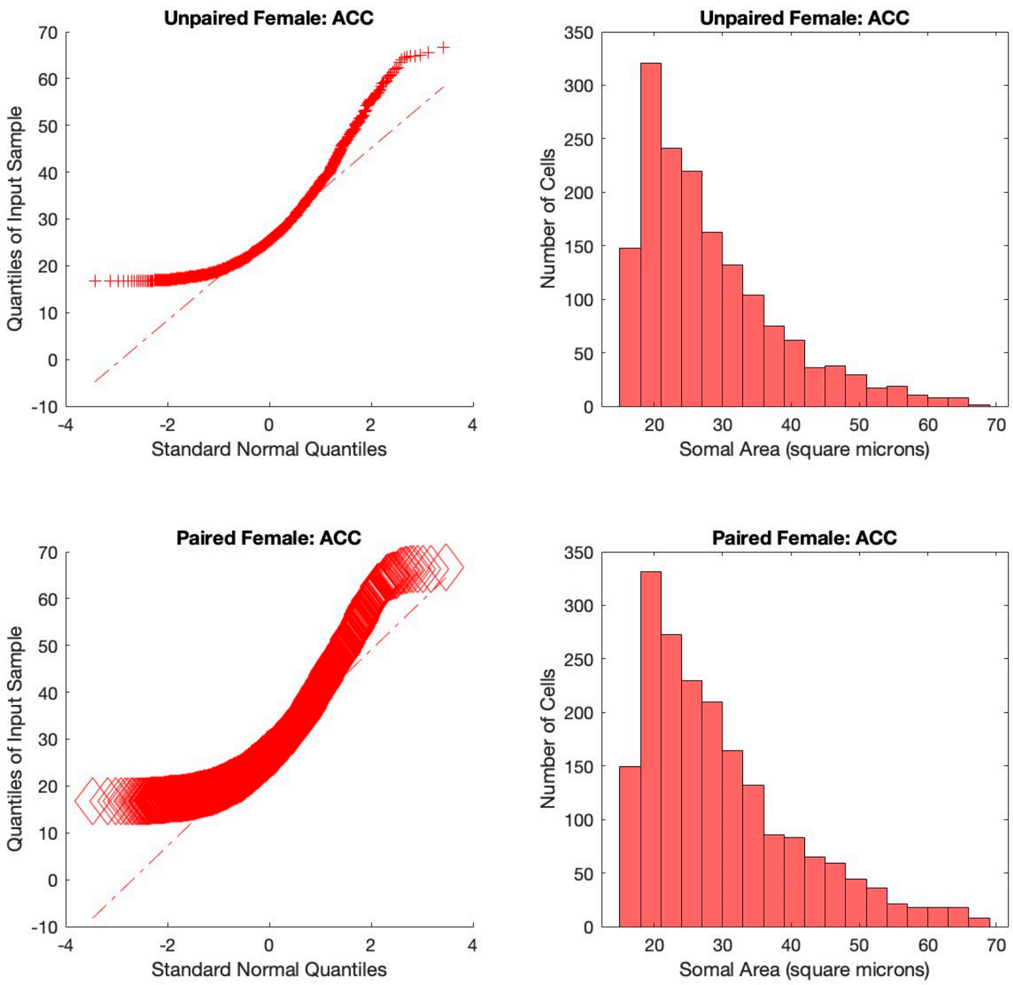

Figure S2. Female ACC distribution visualizations. Q-Q Plots (Left) and Histograms (Right) produced as visualizations of the distributions of microglia soma areas in the anterior cingulate cortex (ACC). Q-Q Plots depict the unequal variance of each distribution compared to a normal distribution as indicated by the diagonal dashed line. The color *red* indicates female microglia. A small cross indicates unpaired microglia. A large diamond indicates paired microglia.

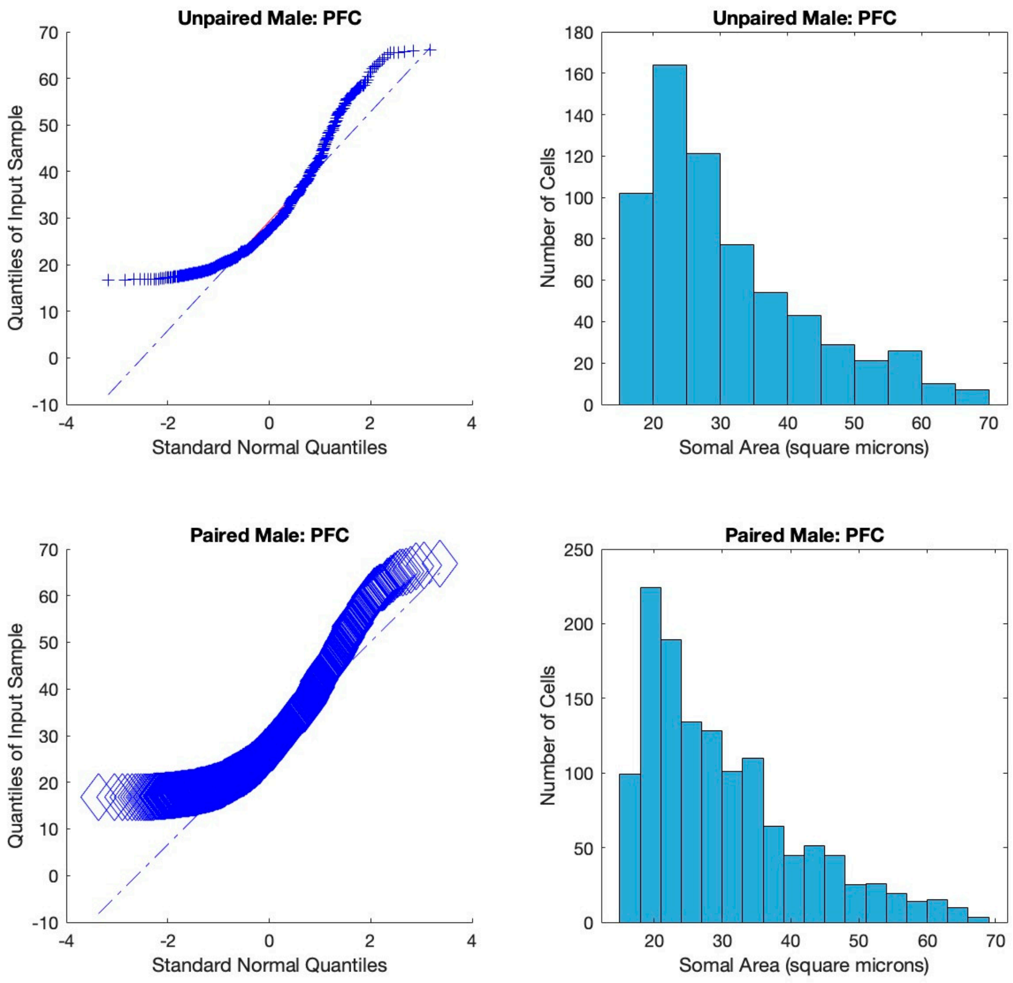

Figure S3. Male PFC distribution visualizations. Q-Q Plots (Left) and Histograms (Right) produced as visualizations of the distributions of microglia soma areas in the prefrontal cortex (PFC). Q-Q Plots depict the unequal variance of each distribution compared to a normal distribution as indicated by the diagonal dashed line. The color *blue* indicates male microglia. A small cross indicates unpaired microglia. A large diamond indicates paired microglia.

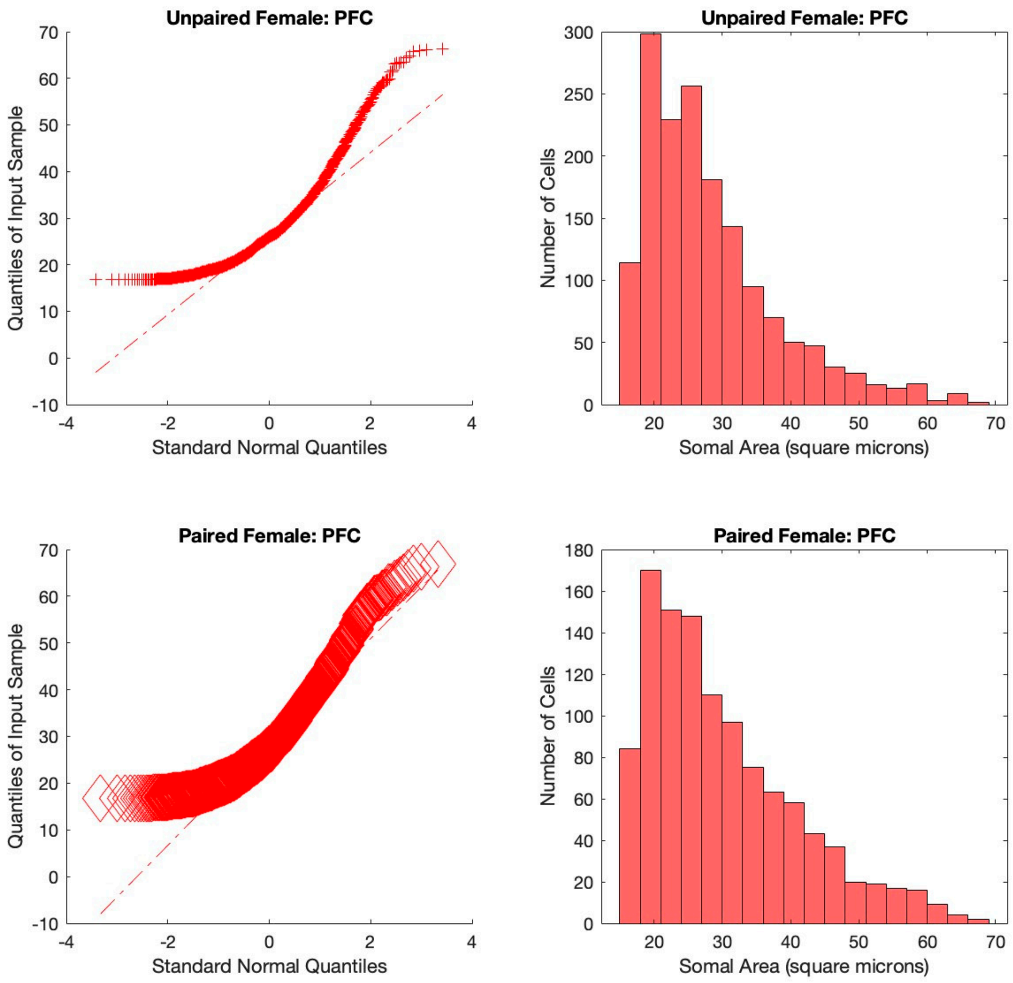

Figure S4. Female PFC distribution visualizations. Q-Q Plots (Left) and Histograms (Right) produced as visualizations of the distributions of microglia soma areas in the prefrontal cortex (PFC). Q-Q Plots depict the unequal variance of each distribution compared to a normal distribution as indicated by the diagonal dashed line. The color *red* indicates female microglia. A small cross indicates unpaired microglia. A large diamond indicates paired microglia.

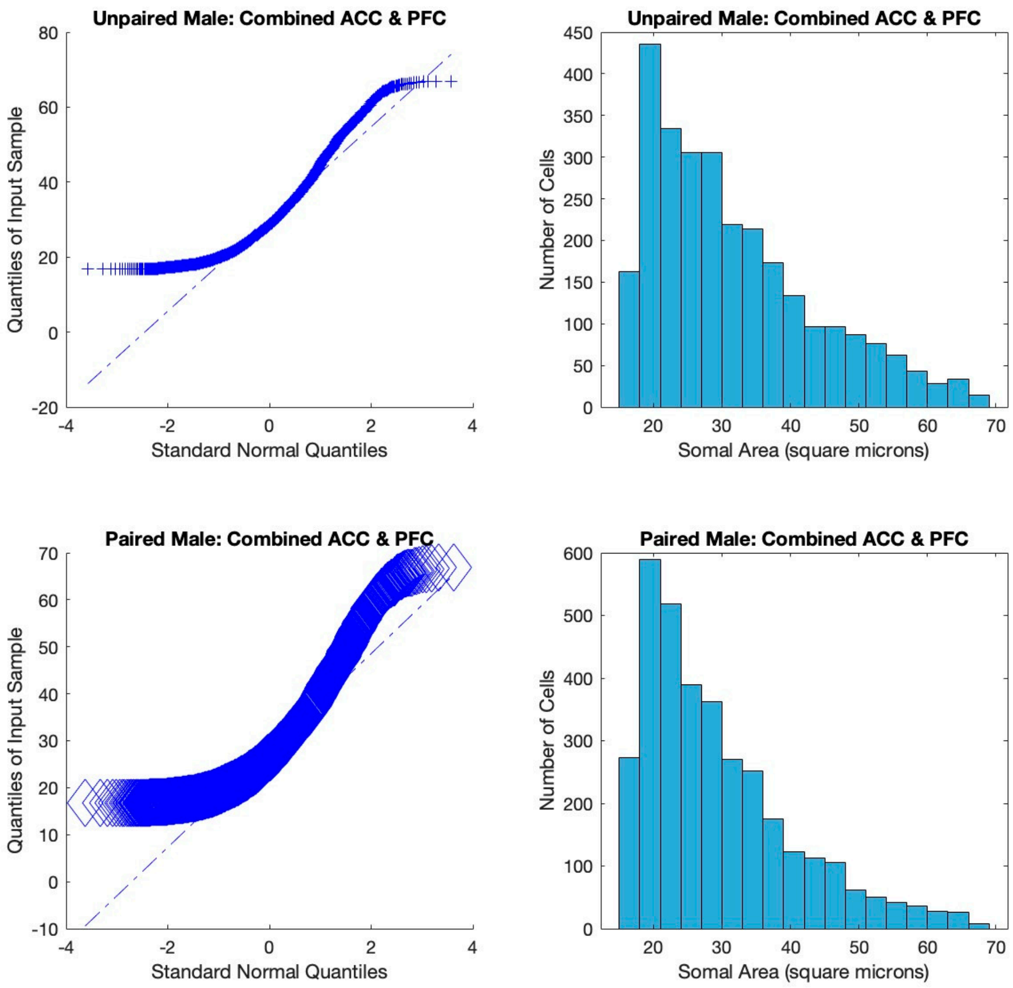

Figure S5. Male combined regions distribution visualizations. Q-Q Plots (Left) and Histograms (Right) produced as visualizations of the distributions of microglia soma areas in the anterior cingulate cortex (ACC) and prefrontal cortex (PFC) combined. Q-Q Plots depict the unequal variance of each distribution compared to a normal distribution as indicated by the diagonal dashed line. The color *blue* indicates male microglia. A small cross indicates unpaired microglia. A large diamond indicates paired microglia.

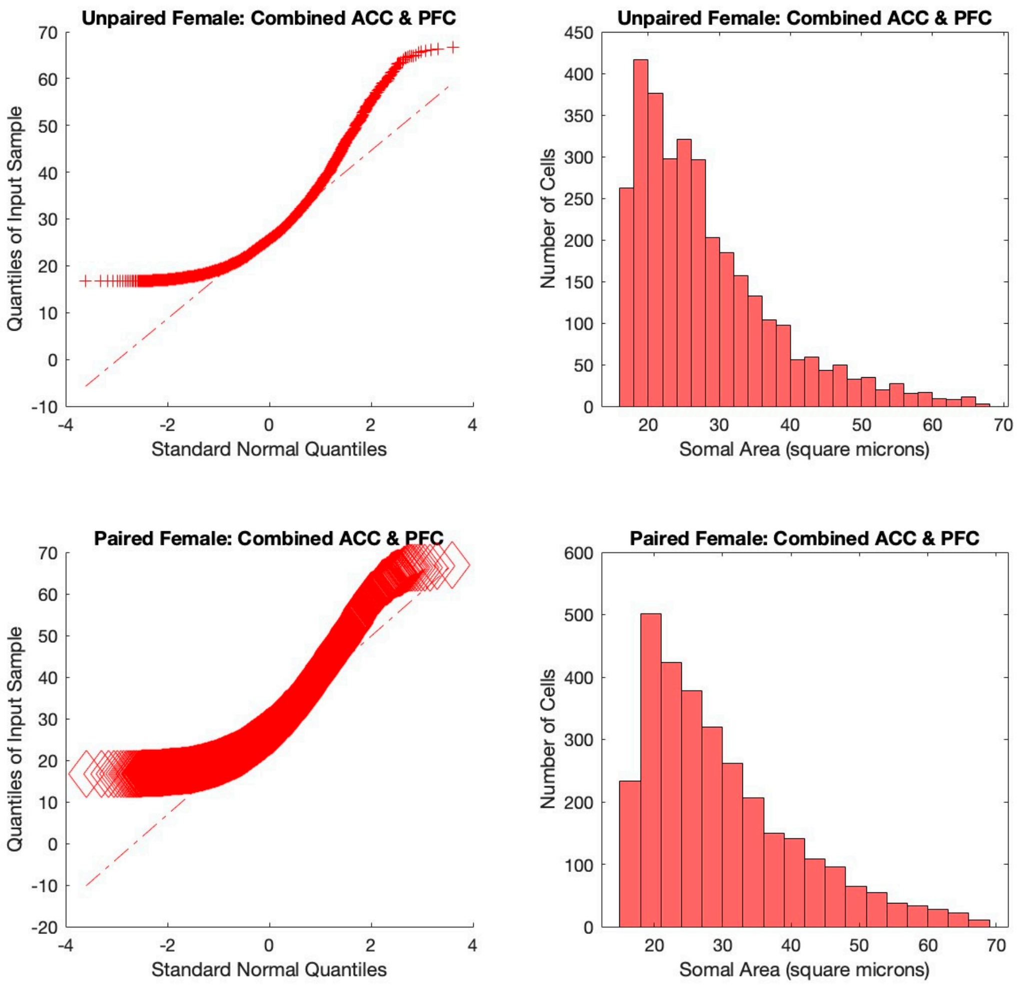

Figure S6. Female combined regions distribution visualizations. Q-Q Plots (Left) and Histograms (Right) produced as visualizations of the distributions of microglia soma areas in the anterior cingulate cortex (ACC) and prefrontal cortex (PFC) combined. Q-Q Plots depict the unequal variance of each distribution compared to a normal distribution as indicated by the diagonal dashed line. The color *red* indicates female microglia. A small cross indicates unpaired microglia. A large diamond indicates paired microglia.

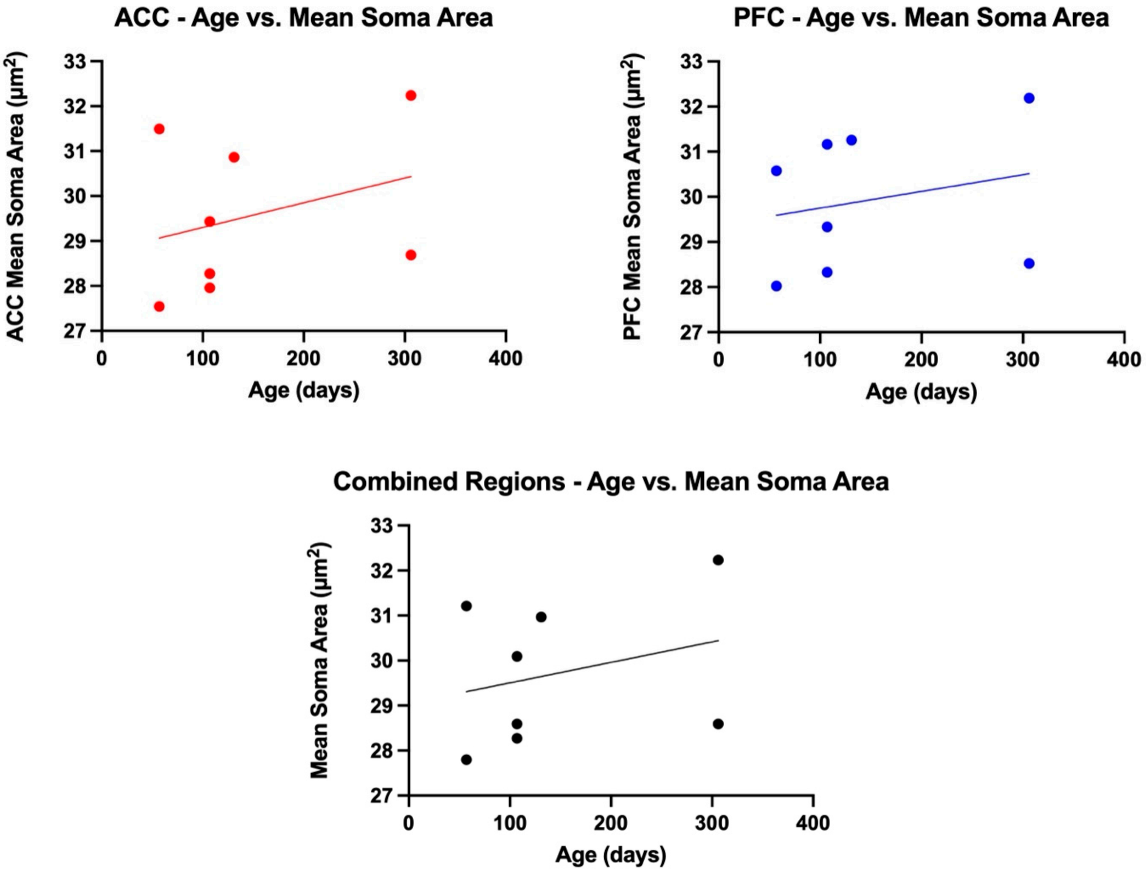

Figure S7. Age does not predict Mean Soma Area. Simple linear regressions of prairie vole age in days versus mean soma area. Each point represents one prairie vole. Red represents data from the anterior cingulate cortex (ACC) only (r^2 = 0.1002). Blue represents data from the prefrontal cortex (PFC) only (r^2 = 0.05664). Black represents data from both regions combined (r^2 = 0.08019).

SUPPLEMENTARY METHODS

Sectioning and Tissue Preparation

Prairie vole brains arrived at the lab having been perfused with 1x Phosphate Buffered Saline (PBS) followed by 4% Paraformaldehyde (PFA) for short-term storage. Upon receiving, brains were stored at 4 degrees Celsius until sectioning. Each subject was sectioned coronally at 30 microns using a freezing microtome, with sections passed into six different rostral-caudal samples per subject. Sections were stored in a six well plate containing 5mL of PBS with 0.05% sodium azide as a preservative per well for long-term storage.

After sectioning, 8 samples (one per subject) were selected for immunohistochemistry (IHC). Samples were placed in tissue carriers (Corning NetWell CLS3479, 24mm diameter, 74µm mesh) using a paintbrush to allow for ease of transferring tissue between reagents. Only in-tact sections anterior to the hippocampal formation were selected for staining. Sections were placed unfolded and flat in the tissue carrier, one at a time, while the carrier was submerged in 5mL of PBS in order to minimize twisting. After tissue loading, each subject was placed in a new six well plate containing 5mL PBS per well to begin rinsing.

Antigen Retrieval

Due to prolonged storage in 4% paraformaldehyde during shipping and storage, antigen retrieval was deemed necessary to remove cross-linked formalins and performed on samples prior to staining. Samples were incubated in 1x Tris-EDTA (TE) Buffer (Fisher BioReagents BP2477-500, pH 7.4) in a water bath at 95-100 degrees Celsius for 40 minutes. Samples were allowed to cool slowly back to room temperature while submerged in the TE Buffer before being moved to PBS for rinsing. Boiling temperatures were necessary due to accidental, excessive storage in PFA (> 2 months). Additionally, sections had been stored at 4 degrees Celsius in PBS prior to antigen retrieval. The combination of 4 degrees Celsius acclimation and excessive fixation produced conditions where boiling the cryosections did not damage the tissue integrity.

Several less harsh antigen retrieval methods were attempted before settling on a boiling water bath. Tissue integrity was confirmed after boiling by comparing samples to those that had not received antigen retrieval.

Free-Floating Immunohistochemistry

The staining protein of interest was the Ionized calcium binding adaptor molecule 1 (Iba-1). Iba-1 is a molecule specific to macrophages and microglia that is involved in the phagocytic and pruning functions of all types of brain-derived macrophages. It is present uniformly throughout the cytoplasm of the cell (Korzhevskii & Kirik, 2016), and has been shown to increase its immunoreactivity following glial cytokine expression/release, meaning it is found in increasing concentrations as microglia reactivity increases (Norden et al., 2016). While not commercially validated in prairie voles, the antibody has been used previously in successful vole studies (Pohl et al., 2021; Donovan et al. 2022).

The tissue underwent Free-Floating Immunohistochemistry (IHC) and a Diaminobenzidine (DAB) reaction according to the reagents specified in the Abcam Rabbit specific HRP/DAB (ABC) Detection IHC Kit (ab64261). Approximately 100-200 microliters of each prepared reagent was diluted in PBS to a total volume of 5mL for each well. 0.3% Triton-X 100 was also added to all blocking steps, the primary incubation, and the secondary incubation for permeabilization. All incubation times were optimized in order to lower costs of additional reagents, and according to the dilutions and number of sections per well as described below.

Endogenous peroxidase activity was blocked with the hydrogen peroxide block in 0.3% Triton X-100 and PBS for 15 min at room temperature on the rocker. This was followed by blocking nonspecific antibody binding with the Protein Block in 0.1% Triton-X 100 and PBS for 30 min at room temperature on the rocker. For primary incubation, tissue was moved to a primary buffer containing Iba-1 polyclonal antibody (Thermo Fisher Invitrogen PA5-27436 Rabbit IgG) (1:500) and left to incubate for 48 hours at 4 degrees Celsius on the rocker. For

secondary incubation, tissue was moved to a secondary buffer containing biotinylated goat anti-rabbit IgG (H+L) (1:25) and left to incubate for 90 minutes at room temperature on the rocker. All antibodies were diluted in 0.3% Triton-X 100 and PBS to a total volume of 5mL per well. All samples were rinsed three to four times in PBS for 15-32 minutes between incubations.

At the conclusion of the secondary incubation and rinsing, tissue was placed in the stock streptavidin peroxidase diluted in PBS to incubate for one hour at room temperature on the shaker. To prepare for the DAB reaction, tissue was rinsed several times in PBS followed by a final rinse in sterile deionized water before being moved to a solution of sterile deionized water containing a 1:25 dilution of 50x DAB Chromogen in DAB Substrate. Well plates were placed on top of a white background while on the rocker to monitor the intensity of brown precipitate. Tissue was left to react with DAB for 10 minutes before being moved to a final rinse in PBS. Sections were then stored in PBS at four degrees Celsius until mounting.

Mounting and Imaging

Sections were mounted out of deionized water onto gel coated slides at a ratio of eight sections per slide using aqueous mounting techniques. Slides were coverslipped using aqueous mounting medium designed for brightfield IHC (Abcam AB64230) and glass coverslips. Images of the ACC and PFC were captured on an LED microscope using a 10x Leica Objective, Basler camera (acA3088-57uc), and Basler imaging software. Regions of Interest (ROIs) were determined using the Allen Mouse Brain Atlas to approximate vole brain regions. Microglia in the anterior cingulate cortex (ACC) and prefrontal cortex (PFC) were counted and traced using the “Analyze Particles” feature in Fiji/ImageJ. PFC was considered as a composite region including the subjects’ Prelimbic Areas and Infralimbic Areas as is standard in rodent literature (Carlén, 2017). Care was taken to exclude meningeal and perivascular macrophages, which also appear as Iba-1 positive cells, from the ROIs. Particle threshold was determined to be 10-80 square microns by measuring the soma diameter of 15 random cells, determining the

minimum and maximum diameter values, and taking the square of each. Data containing soma area and ROI area for each image were exported and analyzed.

Statistical Analyses

All statistical analyses were completed using MATLAB R2023a. Independent samples t-tests, one-way analyses of variance (ANOVA), and two-way ANOVAs were completed to test for statistically significant differences in soma size between microglia in males and females and between microglia in paired and unpaired voles. Simple linear regressions were performed to determine whether vole age predicted mean soma area.
